# Assessing Ethiopia's surgical capacity in light of global surgery 2030 initiatives: Is there progress in the past decade?

**DOI:** 10.1016/j.sopen.2024.03.015

**Published:** 2024-03-28

**Authors:** Cherinet Osebo, Jeremy Grushka, Dan Deckelbaum, Tarek Razek

**Affiliations:** a McGill University Health Centre, Centre for Global Surgery, Department of Surgery, Montreal General Hospital, Montreal, Quebec, Canada; b Hargelle Hospital, Emergency Surgery and Obstetrics Unit, Hargelle, Ethiopia

**Keywords:** Ethiopia, Surgical access, Bellwether procedures, Saving lives through safe surgery, Lancet commission on global surgery

## Abstract

**Background:**

Surgical, anesthetic, and obstetric (SAO) care plays a crucial role in global health, recognized by the World Health Organization (WHO) and The Lancet Commission on Global Surgery (LCoGS). LCoGS outlines six indicators for integrating SAO services into a country's healthcare system through National Surgical Obstetrics and Anesthesia Plans (NSOAPs). In Ethiopia, surgical services progress lacks evaluation. This study assesses current Ethiopian surgical capacity using the LCoGS NSOAPs framework.

**Methods:**

We conducted a narrative review of published literature on critical LCoGS NSAOPs metrics to extract information on key domains; service delivery, workforce, infrastructure, finance, and information management.

**Results:**

Ethiopia's surgical services face challenges, including a low surgical volume (43) and a scarcity of specialist SOA physicians (0.5) per 100,000 population. Over half of Ethiopians reside outside the 2-hour radius of surgery-ready hospitals, and 98 % face surgery-related impoverished expenditures. Lacking the LCoGS-recommended SOA reporting systems, approximately 44 % of facilities exist for handling bellwether procedures. Despite the prevalence of essential surgeries, primary district hospitals have limited operative infrastructures, resulting in disparities in the surgical landscape. Most surgery-ready facilities are concentrated in cities, leaving Ethiopia's 80 % rural population with inadequate access to surgical care.

**Conclusion:**

Ethiopia's surgical capacity falls below LCoGS NSOAPs recommendations, with challenges in infrastructure, personnel, and data retrieval. Critical measures include scaling up access, workforce, public insurance, and information management to enhance SAO services. Ethiopia pioneered in Sub-Saharan Africa by establishing Saving Lives Through Safe Surgery (SaLTS) in response to NSOAPs, but progress lags behind LCoGS recommendations.

## Highlights

• Ethiopia's SAO care challenges: low surgical volume, few specialists.
• Lacking surgical tracking systems hinders SAO service evaluation.
• Ethiopia struggles to align with global surgery recommendations.
• Surgical services burden Ethiopians, driving many into poverty.
• Investing in district hospitals is crucial for enhancing SAO access.

## Key messages

Despite Ethiopia's pioneering efforts in adopting NSOAP packages through SaLTS initiatives in Sub-Saharan Africa, there's an urgent need for comprehensive strategies to accelerate the expansion of surgical access and align with global surgery standards.

## Introduction

Surgery is an integral and irreplaceable component of healthcare [1]. Access to quality SAO care is a fundamental right to health, integral to achieving universal health coverage (UHC) on a global scale [2]. Nevertheless, a significant discrepancy exists between the demand and access to surgical services in low- and middle-income countries (LMICs) [3]. The 2015 LCoGS report catalyzed global surgery advocacy, emphasizing challenges for five billion people accessing SAO care. It underscores the need for 143 million additional surgical procedures annually [4]. In LMICs, comprising 40 % of the global population but contributing <5 % to global surgeries, disparities in access are notably pronounced, especially in Sub-Saharan Africa (SSA) [3,5].

In SSA, the prevalence of surgical conditions is marked by urgent and essential procedures that demand immediate attention, contrasting with developed countries where 80 % of surgeries are elective [6,7]. The study underscores that limited access to medical care contributes to 10 % of premature deaths in the Western world, while in resource-limited settings, inadequate access to surgical interventions leads to poor outcomes, emphasizing the crucial role of surgery in treating life-threatening conditions, preventing premature deaths, and improving longevity [4,8]. Enhancing surgical access in low-income countries, such as Ethiopia, is crucial for increasing life expectancy and improving overall well-being [9,10]. An SSA country reported common major procedures, including cesarean sections, laparotomies, and appendectomies [11]. Despite a rising burden of conditions requiring urgent interventions, evaluations consistently uncover shortages in surgery-ready infrastructure, workforce, services, and supplies [12]. Addressing these significant disparities and improving access to emergency and essential surgical care is crucial for reducing the global burden of disease and could prevent an estimated 1.5 million deaths annually in LMICs [13].

Recognizing the imperative for improved surgical care, the LCoGS proposed the establishment of NSOAPs across five critical domains: service delivery, infrastructure, workforce, financing, and information management [4]. As a proactive response, Ethiopia, a low-income East African country, is at the forefront among other SSA nations, pioneering efforts by adapting NSOAPs through Saving Lives Through Safe Surgery (SaLTS) initiative. Ethiopia designates SaLTS as a national priority to enhance SOA services [10,14]. The country's healthcare system is structured with primary healthcare units, general and specialized hospitals, and private facilities [15]. Ethiopia employs the WHO's Service Availability and Readiness Assessment (SARA) tool for national surgical planning, enabling interfacility comparisons [16,17]. Initiated in 2008, healthcare reforms in Ethiopia underscore decentralization and collaboration between federal and regional health officials; nevertheless, disparities in access to essential and emergency surgery persist across Ethiopian regions [15,18]. Surgery plays a crucial role in achieving Ethiopia's 2030 health policy, aligning closely with the overarching goal of reducing poverty and minimizing preventable premature deaths through equitable resource allocation [14].

While Ethiopia has led in adopting the LCoGS NSOAP through SaLTS initiatives in SSA, there is currently a dearth of published papers assessing surgical service progress post-SaLTS implementation. This study bridges this gap by presenting the first comprehensive assessment of Ethiopia's surgical capacities across the mentioned five domains. The evaluation of progress utilizes the LCoGS NSOAP framework and involves a thorough narrative review of both scientific and grey literature.

## Methods

This review was conducted following the PRISMA–ScR guidelines: PRISMA Extension for Scoping Reviews (PRISMA-ScR): Checklist and Explanation [19]. We opted to conduct a narrative review considering the broadness of the research questions with broader general topic overviews.

### Search strategy

We conducted a narrative review of published articles on surgical capacity in Ethiopia related to LCoGS indicators. We enlisted the assistance of a research librarian at the McGill University Health Centre to aid our search of the MEDLINE (Ovid interface), Embase (Ovid interface), and Global Health (Ovid interface) databases (Appendix 1). The search strategy included the following words “Ethiopia*” “Surg*” OR “Anesthe*” OR “Obstetr*” OR “Catastrophic expenditure*” OR “Impoverished expenditure*” OR “Lancet Commission on Global Surgery*” AND “Bellwether Surgery.” The search was limited to articles published between January 1st 2000, to October 14th 2022, in the English language. We selected articles from peer-reviewed publications in global surgery. Additionally, the reference lists of these publications were searched for additional relevant articles related to surgical, obstetric, and anesthesia care.

### Eligibility criteria

Articles were included based on their direct correlation to LCoGS metrics and Ethiopia's surgical capacity, delivery, and challenges. Articles that failed to satisfy Ethiopian surgical systems were excluded. Studies in relevant domains—service delivery, workforce, infrastructure, information management, finance, and outcomes—had data and significant findings compiled. We further reorganized each Lancet Commission indicator as preoperative, perioperative, and postoperative for study simplicity. The *preoperative* (*Infrastructures and Surgical Volume*) metrics include 80 % of the population within 2-hour travels to hospitals capable of conducting the three bellwether procedures—a laparotomy, cesarean delivery, and open long-bone fracture management, and the availability of 20 SAO providers for a 100,000 population. *Perioperative* (*Surgical Services and Information Management*) metrics include performing 5000 surgeries per 100,000 population annually and establishing a nationwide surgical tracking system. *Postoperative* (*Finances*) metrics cover 100 % protection against impoverishing and catastrophic healthcare expenditures caused by surgery [4].

### Study selection and data extraction

CO and TR independently screened the titles and abstracts of the identified publications for relevance. The reviewers discussed the included articles for incongruencies and reached a consensus. The same authors then read the full-text articles to identify those that met the eligibility criteria. Articles that passed the two screening stages were then charted for relevant data.

## Results

**Fig. 1:**
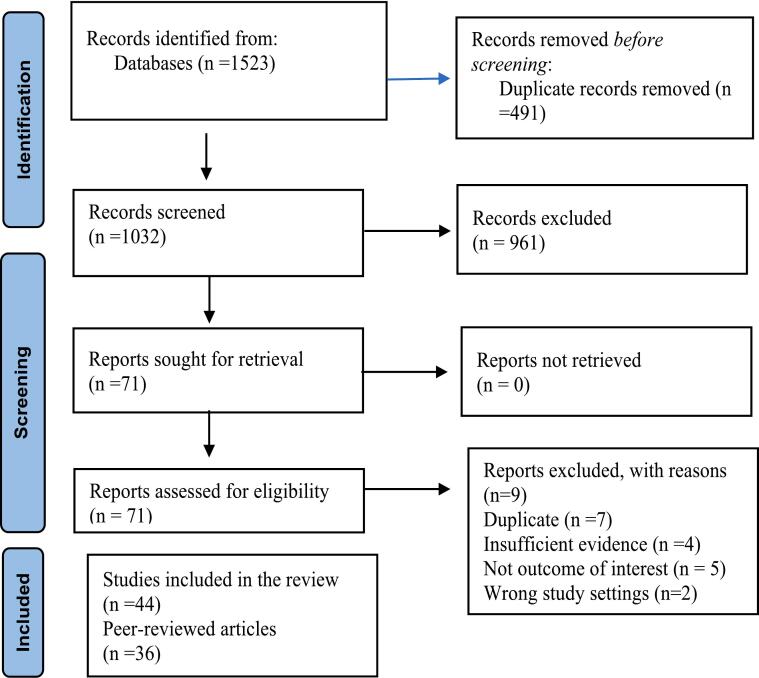
PRISMA flow chart of the search and screening process.

Fig. 1 highlights the process of identifying and selecting the articles included in this review. Through MEDLINE (Ovid interface), Embase (Ovid interface), and Global Health (Ovid interface), we identified 1523 citations. After completing all search strategies, 1032 records underwent title and abstract screening, of which 71 articles were kept undergoing full-text screening. After both stages of screening, 44 articles were included in this review. Of the included articles, 11 were relevant to surgical infrastructure, 12 to workforce, 14 to service delivery, 5 to finance, and 2 to information management. Some of these articles were relevant to multiple domains. Table 1 presents a summary of the narrative review's findings.

**Table 1:** Ethiopia's estimates for Lancet Commission on global surgery indicator.

Parameter	Indicator	aLCoGS 2030 Goal	Ethiopia's estimates
**Preoperative** Infrastructures	Access to essential surgical care	80 % of people within 2 h of a facility capable of performing bellwether procedures^a^	≤50 % [20]
	Surgical workforce density	20/100,000 population^a^	0.54^**?**^ [49] 5.19^**?**^ [21]
**Perioperative** Surgical Services	Surgical volume	5000/100,000 population^a^	43^**?**^ [42] 192^**?**^ [14]
	Cesarean section	10–15 %^b^	0.6 [28] 3.5^**?**^ [29]
	Surgical safety checklist	100 %^b^	61% [32]
Information management	POMR system	100 % national POMR tracking system^c^	No
**Postoperative** Finances	Risk of catastrophic expenditure	100 % protection^a^	15 %^**?**^ [36] 37 %^**?**^ [37]
	Risk of impoverishing expenditure	100 % protection^a^	2 % [49]

The table structure is adapted from Shirley and colleague [50].

^**?**^ indicates a range of values found in the review process, illustrating the variability in Ethiopia's current surgical capacity.

a Lancet and Word Development Indicators on surgical metrics [4,49].

b World Health Organization health indicators [29].

c POMR, perioperative mortality rate. No POMR tracking system in Ethiopia.

### Preoperative indicators

#### Infrastructures

Eleven studies in Ethiopia assessed surgical infrastructure, emphasizing the need for equitable access to facilities capable of performing essential bellwether procedures, such as cesarean sections, laparotomies, and open fracture management, in line with LCoGS metrics. These hospitals should be accessible within a 2-hour travel radius for surgical patients [4]. According to Ouma et al., the majority of Africans, averaging around 80 %, reside within 2 h of surgery-ready facilities, however, for over 50 % of Ethiopians, accessing critical care requires traveling beyond this two-hour threshold [20]. In Ethiopia, surgical patients travel 5, 11, 28.4, and 21.3 h to access services in primary, general, specialized, and private hospitals, respectively [21].

Ethiopian SaLTS reported that 44 % of hospitals offer surgical services, likely reflecting the overall prevalence of surgery-ready hospitals [14]. In a study by Meshesha et al., among 172 health facilities examined, 5.2 % were health center operating room (OR) blocks, 44.8 % were primary hospitals, 22.1 % were general hospitals, 9.3 % were specialized hospitals, and 18.6 % were private hospitals [21]. Regarding supplies, the Ethiopian Hospital Assessment Tool showed that 33 % of facilities lack continuous oxygen, 76 % face interrupted electricity, and 59 % have unreliable running water. Baseline audits across 28 Ethiopian facilities revealed only 29 % had a reliable X-ray machine, and 25 % had a functional ultrasound [10,14] The 2020 Surgical Readiness report indicated scores of 66 %, 47 %, and 53 % for primary, general, and specialized hospitals, respectively, on SARA tracer items for essential surgical services [22]. Among the surveyed hospitals, a significant challenge emerged concerning pulse oximeters, with only 63 % of hospitals having one for each operating room, while the remaining 37 % of hospitals reported some pulse oximetry availability, though not for every operating room [23]. This highlights a disparity in the accessibility of infrastructures across Ethiopian healthcare facilities.

### Workforce

Twelve studies in Ethiopia have assessed surgical workforce density; however, none of them reached an exact number of workforces (Table 1). The LCoGS recommends a minimum of 20 SAO specialists per 100,000 populations. The World Bank estimated in 2016 that there were 0.54 personnel per 100,000 population [24]. A 2022 study [21], that surveyed 172 facilities and revealed that 2312 healthcare professionals were found, including 336 surgeons, 364 anesthesiologists and anesthesia care providers, and 165 obstetricians in specialized hospitals. General hospitals had 123 surgeons, 157 anesthesiologists/anesthesia care providers, 76 obstetricians, and 100 integrated emergency surgical officers (IESOs)— nonphysician surgeons. Primary hospitals had 47 surgeons, 126 anesthesiologists/anesthesia care providers, 16 obstetricians, and 194 IESOs. Overall surgical workforce ratios were 7.5/100,000 in primary hospitals, 1.15/100,000 in general hospitals, and 1.31/100,000 in specialized hospitals, averaging an Ethiopian workforce of 5.19/100,000 population. Obtaining accurate data on the surgical workforce in Ethiopia is challenging.

Another study revealed a wide range in the physician-to-population ratio across Ethiopian regions (1:4715 to 1:107,602). On average, hospitals have 1–2 operating rooms, 4.2 surgeons, 1 gynecologist, and 4.5 anesthesia providers [23]. Notably, >56 % of general surgeons are concentrated in cities, leaving other regions, despite comprising over 95 % of the population, with fewer surgeons [25]. Between 1985 and 2013, 324 general surgeons graduated from Ethiopia, and 327 graduated from Cuba. However, a significant brain drain issue is evident, as many Ethiopian graduates from Cuba reside outside Ethiopia, posing challenges in retaining a skilled workforce [26].

### Perioperative indicators

#### Surgical services

Fourteen studies explored surgical service delivery in Ethiopia, but a systematic evaluation of the national capability for safe SOA care is lacking. The LCoGS 2030 targets 5000 surgeries per 100,000 population annually [4]. However, accurate data on surgical volume in Ethiopia remains fragmented (Table 1). In 2016, the World Bank reported 43 surgeries per 100,000, but by 2019, Kifle et al. indicated a significant tenfold increase to 465 surgeries per 100,000 [24,27]. The 2020 SaLTS recorded 221,260 surgeries, equivalent to 192 per 100,000 [14]. These diverse data reveal fluctuations in surgical volumes.

Markedly, the Ethiopian Ministry of Health encourages cesarean delivery as a preventive measure, showcasing excellence in surgical capacity. Cesarean rates in Ethiopia rose from 0.6 % in 2008 to 3.5 % in 2016 [28,29]. In 2022 [21], a large study identified 69,717 surgical procedures, revealing bellwether procedures in primary hospitals: 3770 cesarean sections, 444 laparotomies, and 14 open fracture management procedures. General hospitals performed 7706 cesarean sections, 1036 laparotomies, and 392 open fracture management procedures, while specialized hospitals conducted 12,673 cesarean sections, 1128 open fracture management procedures, and 1162 laparotomies. Private hospitals contributed 2198 cesarean sections, 706 laparotomies, and 785 open fracture management procedures. Among procedures, emergency procedures related to trauma or obstetrics accounted for 54.3 % of cases, varying from 34.9 % to 82.6 %, with higher rates completed in city centers [23]. Limited surgical supplies in certain hospitals constrain them to addressing emergencies. For instance, general surgeons in district hospitals may lack expertise for specific cases, such as thoracic surgery, primarily handling tasks like occasionally placing chest tubes [21].

Hospitals encounter challenges in achieving maximum surgical volumes, marked by extended waiting times and referral burdens to higher centers. Average pre-admission waiting times for essential surgical care vary from 9.68 days in primary hospitals, 37.6 days in general hospitals, 35.9 days in specialized hospitals, and 1.42 days in private hospitals. The study analyzed 8584 surgical referrals, mainly from primary healthcare units (3956) and public primary hospitals (3540). Referral reasons include a shortage of skilled professionals (30 %) and insufficient equipment (22 %), with 50 % attributed to factors like supply, beds, blood, investigations, finances, and the absence of an ICU [21,23].

#### Information management

Efficient surgical information management is crucial for quality improvement, but Ethiopia lacks sufficient studies on SOA information management, with only two identified. The national tracking systems, LCoGS-recommendation, is currently absent in Ethiopia, relying on incomplete and illegible sources like admission records and operative logbooks [4,22]. The Ethiopian District Health Information Systems (DHIS-2) reported a POMR of 1.4 % in 2020 [30].

Regarding safety and quality, several attempts were made to monitor surgical-site infections (SSI) and surgical safety checklists (SSC) in Ethiopia. In 2012 Chao et al. reported an SSI rate of 1 % [23]. After almost a decade, the 2020 DHIS-2 reported a rate of 1.2 % [30], while the meta-analysis for the same year revealed a 9.8 % SSI rate [31], showcasing discrepancies likely attributable to a shortage of tracking systems. Compliance with SSC in Ethiopian hospitals, integrated into the surgical care strategy for patient safety, was estimated at 61 % in recent studies, while DHIS-2 reported 81 % [30,32].

### Postoperative indicators

#### Finance

Five studies explored the finances of surgical care in Ethiopia, focusing on protection against impoverished expenditure (IE) and catastrophic health expenditure (CHE), crucial LCoGS indicators for surgical patients. By 2030, SOA care could cost the global economy $12.3 million, causing 81 million people to face CHE, with an additional 48.5 million incurring non-medical costs [4,33]. A study from Papua New Guinea found a simple appendectomy costs between $11,300 and $13,300, unaffordable for patients [34].

Ethiopian surgical patients incurred US$204 in medical expenses and US$611 in non-medical costs, including transport, food, and lodging [35]. According to World Bank estimates [36], 98 % of Ethiopians are at risk of IE due to surgery, leaving only 2 % protected, defined as out-of-pocket (OOP) payments driving people into extreme poverty. Furthermore, 85 % face the risk of CHE, with only 15 % protected, exemplified by direct OOP payments exceeding 10 % of the household's annual income. Similar studies estimated the risk of CHE in surgical patients was 62.7 %, indicating that 37.3 % were protected (Table 1) [37].

## Discussion

The review highlights priority policy areas for improving Ethiopia's surgical system. Despite limited specific SOA care data, a comprehensive literature review provides significant evidence. Local capacity assessments like SARA and SaLTS have informed robust LCoGS NSOAP pathways to enhance healthcare structures [14]. SaLTS, a national flagship, aims to enhance access to safe, essential, and emergency SOA care. Success aspects include strong government leadership, utilization of existing learning systems, recognition of partnerships, engagement of stakeholders, the definition of locally relevant care packages, and enhancing and scaling up nationally based on early learning experiences [10]. The WHO recommends evidence-based priority setting, exemplified by Ethiopia's SaLTS initiatives. This review's data, including SaLTS establishments, were summarized into policy briefs for evidence-informed priority setting during the SaLTS development. Despite Ethiopia's pioneering efforts in SSA, assessing SaLTS progress using LCoGS NSOAP metrics is scarce. The following sections detail how the review results informed priority setting in Ethiopian SOA care.

### Preoperative

Equitable access to surgery-ready facilities in Ethiopia, a vital metric targeting 80 % accessibility within 2 h by 2030, faces challenges. Surgical patients, on average, travel 28 h to reach specialized hospitals for critical care, underscoring substantial disparities in location, transport, and infrastructure availability. Transport challenges in surgical emergencies frequently result in delayed presentations and increased complications, significantly impacting hemorrhagic mortalities from delayed obstetric and trauma emergencies [21,23]. Currently, 44 % of bellwether hospitals serve the 117 million Ethiopian population, prompting efforts to double this number to 80 % by 2025 [14]. However, achieving this target is challenging given the current COVID-19 pandemic and Ethiopian political situation [38]. To date, in Ethiopia, the exact number of surgery-ready hospitals capable of providing essential and emergent surgeries is unclear.

Despite efforts to increase medical service accessibility in impoverished rural nations, maternal care barriers persist. In 2010, out of the 3 % of pregnant women who underwent emergency cesarean sections, 20 % were performed for urban women, while only 0.5 % were performed for the poorest rural females, where over 80 % of the country's population dwells [28]. Existing challenges were further evidenced by studies indicating that merely 61 % of 18 hospitals in two large Ethiopian regions had only one functional operating room, with some district hospitals lacking any [10,39]. To address gaps, the government has allocated funds to renovate 370 operating rooms, with 80 completed. An additional 420 operating rooms are under construction in health centers, benefiting underserved rural communities, supported by a $50 million fund for procuring equipment [10]. SafeSurgery2020, through SaLTS, invests in improving oxygen access, planning to construct two oxygen plants in referral hospitals, aligned with the national roadmap and leveraging successful models from other African countries [10,40].

The review reveals a concerning shortage of SOA workforce in Ethiopia, with figures ranging from 0.53 to 5.2 per 100,000 population, indicating data discrepancies [21,24]. Uneven distribution is evident, with 38 % of surgical subspecialists concentrated in urban areas, while 87 % of district primary hospitals lack any SOA personnel [27,39]. This shortage and imbalance impact patient care, satisfaction, and the overall economy. Expanding SOA residency programs can address workforce challenges, with proposed solutions including the design of rural practice pipelines for medical students and addressing infrastructure deficiencies [23,41]. Strategies to discourage emigration and retain Ethiopian surgeons are also crucial [26]. Hospital-based training, following the WHO Global Code of Practice, has shown success in retaining graduates in rural areas, exemplified by the Pan-African Academy of Christian Surgeons at Sodo Christian Hospital in Ethiopia [25]. University-based training programs remain pivotal in nurturing academic SOA specialists, driving surgical education, research, and innovation in the country [25,26]. The health systems strengthening approach seeks to enhance rural workforce conditions by supporting a functional regional SOA hub, focusing on improving the supply chain, staffing, and infrastructure for sustainable and effective healthcare.

### Perioperative

The review underscores a substantial shortage of surgical procedures in Ethiopia, ranging from 43 to 192 procedures per 100,000, with the accurate figure likely falling in the middle [14,42]. Ethiopia is projected to fall short of its goal of 5000 surgeries per 100,000 population by 2060–2070 [43]. A study revealed that 46 % of bellwether procedures occurred in higher hospitals in cities, with only 17 % taking place in district primary hospitals; however, LCoGS mandates all primary hospitals to perform essential life-saving surgeries, showcasing significant disparities in access to care [21]. Consequently, SaLTS aims to enhance surgical service delivery across all healthcare levels, focusing on upgrading health centers for major and emergency surgical and obstetric procedures in rural areas and empowering district hospitals for more complex surgeries [4,10,14].

Despite WHO's recommendation of evenly distributed cesarean section rates between 5 % and 15 %, Ethiopian urban hospitals perform the majority, leaving limited access for rural communities [29,44]. Maternal mortalities decreased from 597 in 2010 to 401 per 100,000 live births in 2017, encouraging more equitable cesarean distribution [30]. The Ethiopian Ministry of Health aims to increase the current 3.6 % cesarean section rate to 10 % to meet life-saving procedure needs, aligning with WHO guidelines [14]. Furthermore, Ethiopia lacks sufficient studies on SOA information management, and while DHIS-2 reports a POMR of 1.4 %, other estimates range from 2 % to 3.3 % [30,45]. The absence of surgical tracking systems hampers obtaining reliable nationwide statistics. In response, Ethiopia's SaLTS initiative seeks to prospective collection and reporting of the six LCoGS indicators [4,10], along with achieving a national mortality rate below 2 % and implementing 100 % SSC at all facilities [14]. SaLTS enhances capacity, addresses referral system inefficiencies, and ensures high-quality service.

### Postoperative

In Ethiopia, surgical patients face substantial financial burdens, incurring an average of US$204 in medical expenses and US$611 in non-medical costs [35]. The World Bank reported only 2 % are protected from IE and 15 % from CHE, but the norm is that 100 % should be protected from surgery-related costs [4,36]. The majority of Ethiopians experience forced out-of-pocket payments, leading to 18 % skipping medical care due to financial constraints, resulting in delayed surgical interventions, leading to poor outcomes [46]. Access to surgery appears limited to those who can afford it, highlighting the need for financial support and policy interventions [37].

To address financial challenges, Ethiopia has implemented policies, including fee waivers for those unable to afford medical care and the introduction of community-based health insurance [47]. Strengthening such programs is crucial to ensuring equitable access to surgical services. Furthermore, the SaLTS governance section prioritizes tracking national budgetary allocations to surgical services, aiming to reduce CHE and IE for patients [10,14]. The initiative advocates for the inclusion of surgical procedures in national health insurance schemes, offering protection to patients and ensuring appropriate reimbursement for health facilities providing surgical services. Investing in surgery has proven economically beneficial, with positive outcomes [48]. Recognizing surgery as an essential part of healthcare [37], SAO care should be integral to Ethiopia's national health system, irrespective of income level.

### Limitations

While this narrative review comprehensively addressed Ethiopia's surgical system, additional research is needed to cover policy areas not included, ensuring SaLTS's comprehensiveness and overall enhancement of the surgical ecosystem. Reliance on scientific and grey literature poses a potential risk of publication bias, and considering the two-decade span, some data may be outdated. The review is grounded in the LCoGS NSOAPs five framework, which may not be a perfect fit for all contexts, as exemplified by Ethiopia's expansion of SaLTS to eight pillars [10]. Ongoing adaptation of the framework is crucial, incorporating lessons learned during SaLTS development. The scarcity of primary research on surgical capacity in Ethiopia limits generalizability, potentially leading to over- or underestimation. The narrative review's broad scope and the limited scientific literature on the topic introduce a possibility of selection and narrative bias. Despite limitations, the review provides insights into the current surgical landscape since adopting the SaLTS program, mirroring other countries' NSOAPs.

## Conclusion

This assessment identifies areas for strengthening Ethiopia's surgical structure, incorporating insights from the SARA measurement and SaLTS assessment tools [10,14,17]. While a comprehensive examination of Ethiopian surgical capacity is lacking, this paper serves as a guide for countries developing NSOAPs, drawing on Ethiopia's experience with SaLTS integration into its national health system. While Ethiopia has been a pioneer among African nations in this regard, our findings underscore shortages in meeting LCoGS metrics, signaling areas for improvement. Policymakers can use this review to shape surgery-related guidelines, and NGOs working on surgical services may gain valuable insights for future development. Challenges facing SaLTS include ensuring consistent implementation of its pillars across all regions, attracting new partners and investors, robustly evaluating early results, and sustaining momentum [10,14]. Moving forward, SaLTS interventions will shape the national scale-up, strengthening SOA care across Ethiopian regions evenly. Strengthening the monitoring and evaluation of SaLTS strategies is crucial for informing evidence-based interventions and implementation policies, benefiting not only Ethiopia but also other LMICs.

## CRediT authorship contribution statement

**Cherinet Osebo:** Conceptualization, Data curation, Formal analysis, Methodology, Writing – original draft, Writing – review & editing. **Jeremy Grushka:** Conceptualization, Data curation, Methodology, Supervision, Writing – review & editing. **Dan Deckelbaum:** Conceptualization, Data curation, Writing – review & editing. **Tarek Razek:** Conceptualization, Data curation, Formal analysis, Methodology, Supervision, Writing – review & editing.

## Declaration of competing interest

The authors declare that they have no conflict of interest.

## Figures and Tables

**Table:** 

Database	Search strategy
MEDLINE Ovid MEDLINE(R) ALL 1946 to October 14, 2022	1 Ethiopia/ 2 Ethiopian surgical capacity or saving life through safe surgery.mp. 3 1 or 2 4 general surgery/ or obstetrics/ or orthopedics/ or traumatology/ 5 laparotomy/ or exp delivery, obstetric/ or open fracture reduction/ 6 surgeons/ or orthopedic surgeons/ or anesthesia/ or anesthesiology 7 (surgeon* or obstetric* or surger* or surgic* or traumatolog* or open fracture* or anesthesia*).mp. [mp = title, book title, abstract, original title, name of substance word, subject heading word, floating sub-heading word, keyword heading word, organism supplementary concept word, protocol supplementary concept word, rare disease supplementary concept word, unique identifier, synonyms] 8 4 or 5 or 6 or 7 9 3 and 8 10 catastrophic expenditure* or finance* or surgery expenses*.mp. 11 3 and 10 12 impoverish* expenditure* or insurance plan* or community health insurance*.mp. 13 3 and 12 14 (lancet commission on global surgery or Lancet Global Surgery or LCoGS 2030) or world health organization or essential surgery.mp. 15 3 and 14 16 (afghanistan or albania or algeria or american samoa or angola or “antigua and barbuda” or antigua or barbuda or argentina or armenia or armenian or aruba or azerbaijan or bahrain or bangladesh or barbados or republic of belarus or belarus or byelarus or belorussia or byelorussian or belize or british honduras or benin or dahomey or bhutan or bolivia or “bosnia and herzegovina” or bosnia or herzegovina or botswana or bechuanaland or brazil or brasil or bulgaria or burkina faso or burkina fasso or upper volta or burundi or urundi or cabo verde or cape verde or cambodia or kampuchea or khmer republic or cameroon or cameron or cameroun or central african republic or ubangi shari or chad or chile or china or colombia or comoros or comoro islands or iles comores or mayotte or democratic republic of the congo or democratic republic congo or congo or zaire or costa rica or “cote d'ivoire” or “cote d' ivoire” or cote divoire or cote d ivoire or ivory coast or croatia or cuba or cyprus or czech republic or czechoslovakia or djibouti or french somaliland or dominica or dominican republic or ecuador or egypt or united arab republic or el salvador or equatorial guinea or spanish guinea or eritrea or estonia or eswatini or swaziland or ethiopia or fiji or gabon or gabonese republic or gambia or “georgia (republic)” or georgian or ghana or gold coast or gibraltar or greece or grenada or guam or guatemala or guinea or guinea bissau or guyana or british guiana or haiti or hispaniola or honduras or hungary or india or indonesia or timor or iran or iraq or isle of man or jamaica or jordan or kazakhstan or kazakh or kenya or “democratic people's republic of korea” or republic of korea or north korea or south korea or korea or kosovo or kyrgyzstan or kirghizia or kirgizstan or kyrgyz republic or kirghiz or laos or lao pdr or “lao people's democratic republic” or latvia or lebanon or lebanese republic or lesotho or basutoland or liberia or libya or libyan arab jamahiriya or lithuania or macau or macao or republic of north macedonia or macedonia or madagascar or malagasy republic or malawi or nyasaland or malaysia or malay federation or malaya federation or maldives or indian ocean islands or indian ocean or mali or malta or micronesia or federated states of micronesia or kiribati or marshall islands or nauru or northern mariana islands or palau or tuvalu or mauritania or mauritius or mexico or moldova or moldovian or mongolia or montenegro or morocco or ifni or mozambique or portuguese east africa or myanmar or burma or namibia or nepal or netherlands antilles or nicaragua or niger or nigeria or oman or muscat or pakistan or panama or papua new guinea or new guinea or paraguay or peru or philippines or philipines or phillipines or phillippines or poland or “polish people's republic” or portugal or portuguese republic or puerto rico or romania or russia or russian federation or ussr or soviet union or union of soviet socialist republics or rwanda or ruanda or samoa or pacific islands or polynesia or samoan islands or navigator island or navigator islands or “sao tome and principe” or saudi arabia or senegal or serbia or seychelles or sierra leone or slovakia or slovak republic or slovenia or melanesia or solomon island or solomon islands or norfolk island or norfolk islands or somalia or south africa or south sudan or sri lanka or ceylon or “saint kitts and nevis” or “st. kitts and nevis” or saint lucia or “st. lucia” or “saint vincent and the grenadines” or saint vincent or “st. vincent” or grenadines or sudan or suriname or surinam or dutch guiana or netherlands guiana or syria or syrian arab republic or tajikistan or tadjikistan or tadzhikistan or tadzhik or tanzania or tanganyika or thailand or siam or timor leste or east timor or togo or togolese republic or tonga or “trinidad and tobago” or trinidad or tobago or tunisia or turkey or turkmenistan or turkmen or uganda or ukraine or uruguay or uzbekistan or uzbek or vanuatu or new hebrides or venezuela or vietnam or viet nam or middle east or west bank or gaza or palestine or yemen or yugoslavia or zambia or zimbabwe or northern rhodesia or global south or africa south of the sahara or sub-saharan africa or subsaharan africa or africa, central or central africa or africa, northern or north africa or northern africa or magreb or maghrib or sahara or africa, southern or southern africa or africa, eastern or east africa or eastern africa or africa, western or west africa or western africa or west indies or indian ocean islands or caribbean or central america or latin america or “south and central america” or south america or asia, central or central asia or asia, northern or north asia or northern asia or asia, southeastern or southeastern asia or south eastern asia or southeast asia or south east asia or asia, western or western asia or europe, eastern or east europe or eastern europe or developing country or developing countries or developing nation? or developing population? or developing world or less developed countr* or less developed nation? or less developed population? or less developed world or lesser developed countr* or lesser developed nation? or lesser developed population? or lesser developed world or under developed countr* or under developed nation? or under developed population? or under developed world or underdeveloped countr* or underdeveloped nation? or underdeveloped population? or underdeveloped world or middle income countr* or middle income nation? or middle income population? or low income countr* or low income nation? or low income population? or lower income countr* or lower income nation? or lower income population? or underserved countr* or underserved nation? or underserved population? or underserved world or under served countr* or under served nation? or under served population? or under served world or deprived countr* or deprived nation? or deprived population? or deprived world or poor countr* or poor nation? or poor population? or poor world or poorer countr* or poorer nation? or poorer population? or poorer world or developing econom* or less developed econom* or lesser developed econom* or under developed econom* or underdeveloped econom* or middle income econom* or low income econom* or lower income econom* or low gdp or low gnp or low gross domestic or low gross national or lower gdp or lower gnp or lower gross domestic or lower gross national or lmic or lmics or third world or lami countr* or transitional countr* or emerging economies or emerging nation?).ti,ab,sh,kf. 17 14 and 16 18 surg* anesthe* or obstetr* and bellwether*.mp. 19 3 and 18 20 4 or 6 or 7 or 19 21 3 and 20 22 3 and 6 23 surgical capacit*.mp. 24 3 and 23 25 16 and 23 26 14 or 20/ 16–23 27 limit 26 to yr = “2000–Current”
Global Health Ovid Global Health 1973 to 2022 Week 07	1 Ethiopian surgical strategy/ 2 Ethiopian surgical capacity or saving life through safe surgery.mp. 3 general surgery/ or obstetrics/ or orthopedics/ or traumatology/ 4 laparotomy/ or exp delivery, obstetric/ or open fracture reduction/ 5 surgeons/ or orthopedic surgeons/ or anesthesia/ or anesthesiology 6 (surgeon* or obstetric* or surger* or surgic* or traumatolog* or open fracture* or anesthesia*).mp. [mp = title, book title, abstract, original title, name of substance word, subject heading word, floating sub-heading word, keyword heading word, organism supplementary concept word, protocol supplementary concept word, rare disease supplementary concept word, unique identifier, synonyms] 7 catastrophic expenditure* or finance* or surgery expenses*.mp. 8 impoverish* expenditure* or insurance plan* or community health insurance*.mp. 9 (lancet commission on global surgery or Lancet Global Surgery or LCoGS 2030) or world health organization or essential surgery.mp. 10 1 or2 or 3 or 4 or 5 or 6 or 7 or 8 or 9 or 10 11 (afghanistan or albania or algeria or american samoa or angola or “antigua and barbuda” or antigua or barbuda or argentina or armenia or armenian or aruba or azerbaijan or bahrain or bangladesh or barbados or republic of belarus or belarus or byelarus or belorussia or byelorussian or belize or british honduras or benin or dahomey or bhutan or bolivia or “bosnia and herzegovina” or bosnia or herzegovina or botswana or bechuanaland or brazil or brasil or bulgaria or burkina faso or burkina fasso or upper volta or burundi or urundi or cabo verde or cape verde or cambodia or kampuchea or khmer republic or cameroon or cameron or cameroun or central african republic or ubangi shari or chad or chile or china or colombia or comoros or comoro islands or iles comores or mayotte or democratic republic of the congo or democratic republic congo or congo or zaire or costa rica or “cote d'ivoire” or “cote d' ivoire” or cote divoire or cote d ivoire or ivory coast or croatia or cuba or cyprus or czech republic or czechoslovakia or djibouti or french somaliland or dominica or dominican republic or ecuador or egypt or united arab republic or el salvador or equatorial guinea or spanish guinea or eritrea or estonia or eswatini or swaziland or ethiopia or fiji or gabon or gabonese republic or gambia or “georgia (republic)” or georgian or ghana or gold coast or gibraltar or greece or grenada or guam or guatemala or guinea or guinea bissau or guyana or british guiana or haiti or hispaniola or honduras or hungary or india or indonesia or timor or iran or iraq or isle of man or jamaica or jordan or kazakhstan or kazakh or kenya or “democratic people's republic of korea” or republic of korea or north korea or south korea or korea or kosovo or kyrgyzstan or kirghizia or kirgizstan or kyrgyz republic or kirghiz or laos or lao pdr or “lao people's democratic republic” or latvia or lebanon or lebanese republic or lesotho or basutoland or liberia or libya or libyan arab jamahiriya or lithuania or macau or macao or republic of north macedonia or macedonia or madagascar or malagasy republic or malawi or nyasaland or malaysia or malay federation or malaya federation or maldives or indian ocean islands or indian ocean or mali or malta or micronesia or federated states of micronesia or kiribati or marshall islands or nauru or northern mariana islands or palau or tuvalu or mauritania or mauritius or mexico or moldova or moldovian or mongolia or montenegro or morocco or ifni or mozambique or portuguese east africa or myanmar or burma or namibia or nepal or netherlands antilles or nicaragua or niger or nigeria or oman or muscat or pakistan or panama or papua new guinea or new guinea or paraguay or peru or philippines or philipines or phillipines or phillippines or poland or “polish people's republic” or portugal or portuguese republic or puerto rico or romania or russia or russian federation or ussr or soviet union or union of soviet socialist republics or rwanda or ruanda or samoa or pacific islands or polynesia or samoan islands or navigator island or navigator islands or “sao tome and principe” or saudi arabia or senegal or serbia or seychelles or sierra leone or slovakia or slovak republic or slovenia or melanesia or solomon island or solomon islands or norfolk island or norfolk islands or somalia or south africa or south sudan or sri lanka or ceylon or “saint kitts and nevis” or “st. kitts and nevis” or saint lucia or “st. lucia” or “saint vincent and the grenadines” or saint vincent or “st. vincent” or grenadines or sudan or suriname or surinam or dutch guiana or netherlands guiana or syria or syrian arab republic or tajikistan or tadjikistan or tadzhikistan or tadzhik or tanzania or tanganyika or thailand or siam or timor leste or east timor or togo or togolese republic or tonga or “trinidad and tobago” or trinidad or tobago or tunisia or turkey or turkmenistan or turkmen or uganda or ukraine or uruguay or uzbekistan or uzbek or vanuatu or new hebrides or venezuela or vietnam or viet nam or middle east or west bank or gaza or palestine or yemen or yugoslavia or zambia or zimbabwe or northern rhodesia or global south or africa south of the sahara or sub-saharan africa or subsaharan africa or africa, central or central africa or africa, northern or north africa or northern africa or magreb or maghrib or sahara or africa, southern or southern africa or africa, eastern or east africa or eastern africa or africa, western or west africa or western africa or west indies or indian ocean islands or caribbean or central america or latin america or “south and central america” or south america or asia, central or central asia or asia, northern or north asia or northern asia or asia, southeastern or southeastern asia or south eastern asia or southeast asia or south east asia or asia, western or western asia or europe, eastern or east europe or eastern europe or developing country or developing countries or developing nation? or developing population? or developing world or less developed countr* or less developed nation? or less developed population? or less developed world or lesser developed countr* or lesser developed nation? or lesser developed population? or lesser developed world or under developed countr* or under developed nation? or under developed population? or under developed world or underdeveloped countr* or underdeveloped nation? or underdeveloped population? or underdeveloped world or middle income countr* or middle income nation? or middle income population? or low income countr* or low income nation? or low income population? or lower income countr* or lower income nation? or lower income population? or underserved countr* or underserved nation? or underserved population? or underserved world or under served countr* or under served nation? or under served population? or under served world or deprived countr* or deprived nation? or deprived population? or deprived world or poor countr* or poor nation? or poor population? or poor world or poorer countr* or poorer nation? or poorer population? or poorer world or developing econom* or less developed econom* or lesser developed econom* or under developed econom* or underdeveloped econom* or middle income econom* or low income econom* or lower income econom* or low gdp or low gnp or low gross domestic or low gross national or lower gdp or lower gnp or lower gross domestic or lower gross national or lmic or lmics or third world or lami countr* or transitional countr* or emerging economies or emerging nation?).ti,ab,sh,kf. 12 surg* anesthe* or obstetr* and bellwether*.mp. 13 surgical capacit*.mp. 14 Or/11–13 15 10 and 14 16 limit 15 to yr = “2000–Current”
Embase Ovid Embase 1974 to 2022 October 14	1 National surgical obstetric and anesthesia care plan/ 2 Ethiopian surgical capacity or saving life through safe surgery.mp. 3 1 or 2 4 general surgery/ or obstetrics/ or orthopedics/ or traumatology/ 5 laparotomy/ or exp delivery, obstetric/ or open fracture reduction/ 6 surgeons/ or orthopedic surgeons/ or anesthesia/ or anesthesiology 7 (surgeon* or obstetric* or surger* or surgic* or traumatolog* or open fracture* or anesthesia*).mp. [mp = title, book title, abstract, original title, name of substance word, subject heading word, floating sub-heading word, keyword heading word, organism supplementary concept word, protocol supplementary concept word, rare disease supplementary concept word, unique identifier, synonyms] 8 4 or 5 or 6 or 7 9 3 and 8 10 catastrophic expenditure* or finance* or surgery expenses*.mp. 11 3 and 10 12 impoverish* expenditure* or insurance plan* or community health insurance*.mp. 13 3 and 12 14 (lancet commission on global surgery or Lancet Global Surgery or LCoGS 2030) or world health organization or essential surgery.mp. 15 3 and 14 16 (afghanistan or albania or algeria or american samoa or angola or “antigua and barbuda” or antigua or barbuda or argentina or armenia or armenian or aruba or azerbaijan or bahrain or bangladesh or barbados or republic of belarus or belarus or byelarus or belorussia or byelorussian or belize or british honduras or benin or dahomey or bhutan or bolivia or “bosnia and herzegovina” or bosnia or herzegovina or botswana or bechuanaland or brazil or brasil or bulgaria or burkina faso or burkina fasso or upper volta or burundi or urundi or cabo verde or cape verde or cambodia or kampuchea or khmer republic or cameroon or cameron or cameroun or central african republic or ubangi shari or chad or chile or china or colombia or comoros or comoro islands or iles comores or mayotte or democratic republic of the congo or democratic republic congo or congo or zaire or costa rica or “cote d'ivoire” or “cote d' ivoire” or cote divoire or cote d ivoire or ivory coast or croatia or cuba or cyprus or czech republic or czechoslovakia or djibouti or french somaliland or dominica or dominican republic or ecuador or egypt or united arab republic or el salvador or equatorial guinea or spanish guinea or eritrea or estonia or eswatini or swaziland or ethiopia or fiji or gabon or gabonese republic or gambia or “georgia (republic)” or georgian or ghana or gold coast or gibraltar or greece or grenada or guam or guatemala or guinea or guinea bissau or guyana or british guiana or haiti or hispaniola or honduras or hungary or india or indonesia or timor or iran or iraq or isle of man or jamaica or jordan or kazakhstan or kazakh or kenya or “democratic people's republic of korea” or republic of korea or north korea or south korea or korea or kosovo or kyrgyzstan or kirghizia or kirgizstan or kyrgyz republic or kirghiz or laos or lao pdr or “lao people's democratic republic” or latvia or lebanon or lebanese republic or lesotho or basutoland or liberia or libya or libyan arab jamahiriya or lithuania or macau or macao or republic of north macedonia or macedonia or madagascar or malagasy republic or malawi or nyasaland or malaysia or malay federation or malaya federation or maldives or indian ocean islands or indian ocean or mali or malta or micronesia or federated states of micronesia or kiribati or marshall islands or nauru or northern mariana islands or palau or tuvalu or mauritania or mauritius or mexico or moldova or moldovian or mongolia or montenegro or morocco or ifni or mozambique or portuguese east africa or myanmar or burma or namibia or nepal or netherlands antilles or nicaragua or niger or nigeria or oman or muscat or pakistan or panama or papua new guinea or new guinea or paraguay or peru or philippines or philipines or phillipines or phillippines or poland or “polish people's republic” or portugal or portuguese republic or puerto rico or romania or russia or russian federation or ussr or soviet union or union of soviet socialist republics or rwanda or ruanda or samoa or pacific islands or polynesia or samoan islands or navigator island or navigator islands or “sao tome and principe” or saudi arabia or senegal or serbia or seychelles or sierra leone or slovakia or slovak republic or slovenia or melanesia or solomon island or solomon islands or norfolk island or norfolk islands or somalia or south africa or south sudan or sri lanka or ceylon or “saint kitts and nevis” or “st. kitts and nevis” or saint lucia or “st. lucia” or “saint vincent and the grenadines” or saint vincent or “st. vincent” or grenadines or sudan or suriname or surinam or dutch guiana or netherlands guiana or syria or syrian arab republic or tajikistan or tadjikistan or tadzhikistan or tadzhik or tanzania or tanganyika or thailand or siam or timor leste or east timor or togo or togolese republic or tonga or “trinidad and tobago” or trinidad or tobago or tunisia or turkey or turkmenistan or turkmen or uganda or ukraine or uruguay or uzbekistan or uzbek or vanuatu or new hebrides or venezuela or vietnam or viet nam or middle east or west bank or gaza or palestine or yemen or yugoslavia or zambia or zimbabwe or northern rhodesia or global south or africa south of the sahara or sub-saharan africa or subsaharan africa or africa, central or central africa or africa, northern or north africa or northern africa or magreb or maghrib or sahara or africa, southern or southern africa or africa, eastern or east africa or eastern africa or africa, western or west africa or western africa or west indies or indian ocean islands or caribbean or central america or latin america or “south and central america” or south america or asia, central or central asia or asia, northern or north asia or northern asia or asia, southeastern or southeastern asia or south eastern asia or southeast asia or south east asia or asia, western or western asia or europe, eastern or east europe or eastern europe or developing country or developing countries or developing nation? or developing population? or developing world or less developed countr* or less developed nation? or less developed population? or less developed world or lesser developed countr* or lesser developed nation? or lesser developed population? or lesser developed world or under developed countr* or under developed nation? or under developed population? or under developed world or underdeveloped countr* or underdeveloped nation? or underdeveloped population? or underdeveloped world or middle income countr* or middle income nation? or middle income population? or low income countr* or low income nation? or low income population? or lower income countr* or lower income nation? or lower income population? or underserved countr* or underserved nation? or underserved population? or underserved world or under served countr* or under served nation? or under served population? or under served world or deprived countr* or deprived nation? or deprived population? or deprived world or poor countr* or poor nation? or poor population? or poor world or poorer countr* or poorer nation? or poorer population? or poorer world or developing econom* or less developed econom* or lesser developed econom* or under developed econom* or underdeveloped econom* or middle income econom* or low income econom* or lower income econom* or low gdp or low gnp or low gross domestic or low gross national or lower gdp or lower gnp or lower gross domestic or lower gross national or lmic or lmics or third world or lami countr* or transitional countr* or emerging economies or emerging nation?).ti,ab,sh,kf. 17 14 and 16 18 surg* anesthe* or obstetr* and bellwether*.mp. 19 3 and 18 20 4 or 6 or 7 or 19 21 3 and 20 22 3 and 6 23 surgical capacit*.mp. 24 3 and 23 25 16 and 23 26 14 or 20/ 16–23 27 limit 26 to yr = “2000–Current”
